# Addition of venetoclax to myeloablative conditioning regimens for allogeneic hematopoietic stem cell transplantation in high-risk AML

**DOI:** 10.1080/07853890.2022.2164610

**Published:** 2023-01-11

**Authors:** Xing-yu Cao, Jia-qi Chen, Hui Wang, Wei Ma, Wei-wei Liu, Fang-fang Zhang, Song Xue, Lei Dong, Ting Liu, Xiao-zhen Zhao, Chan-chan Liu, Xin Xu, Yang He, Lei Wang, Jian-ling Wang

**Affiliations:** a Department of Bone Marrow Transplant, Hebei Yanda Lu Daopei Hospital, Langfang, China; b Division of Laboratory Medicine, Hebei Yanda Lu Daopei Hospital, Langfang, China; c Department of Clinical Diagnosis, Hebei Yanda Lu Daopei Hospital, Langfang, China; d Department of Clinical Pharmacology, Hebei Yanda Lu Daopei Hospital, Langfang, China; e HLA Typing Laboratory, Hebei Yanda Lu Daopei Hospital, Langfang, China

**Keywords:** Acute myeloid leukemia (AML), allogeneic hematopoietic stem cell transplantation (allo-HSCT), venetoclax, myeloablative conditioning (MAC)

## Abstract

**Background:**

Venetoclax monotherapy is an effective option for patients with acute myeloid leukemia (AML). Venetoclax has also been used in non-myeloablative conditioning allogeneic hematopoietic stem cell transplantation (allo-HSCT) for high-risk AML with a tolerable toxicity profile. However, the efficacy and safety of a venetoclax-containing myeloablative conditioning (MAC) allo-HSCT regimen for high-risk AML have not been evaluated.

**Objective:**

To evaluate the safety and efficacy of a MAC regimen containing venetoclax for high-risk AML.

**Study design:**

From 25 February 2021 to 4 September 2022, a total of 31 patients with high-risk AML who underwent allo-HSCT and a MAC regimen with venetoclax were analyzed.

**Results:**

At the time of transplantation, 21 patients were in first complete remission (CR1), 4 were in a second complete remission (CR2), and 6 in non-remission (NR). Twenty-four patients (77.4%) were minimal residual disease (MRD)-positive before transplant. The FLT3-ITD gene mutation was present in 51.6% of patients. NUP98 rearrangement, MLL rearrangement or MLL-PTD and DEK::CAN fusion genes were found in 5 (16.1%), 7(22.6%) and 2 (6.5%) patients, respectively. Twenty-nine (93.6%) patients underwent haploidentical allo-HSCT. The median follow-up time was 278 days (range: 52–632 days). The 100-day cumulative incidence of grade 3 to 4 acute graft-versus-host disease (aGVHD) was 16.1% (95%CI, 7.2–36.0%). The 180-day cumulative incidence of moderate to severe chronic graft-versus-host disease (cGVHD) was 7.1% (95%CI, 1.9–26.9%). Cumulative incidence of 100-day cytomegalovirus (CMV) viraemia and 100-day Epstein-Barr virus (EBV) viraemia was 61.6% (95%CI, 46.5–81.4%) and 3.2% (95%CI, 0.4–22.2%), respectively. The 600-day overall survival (OS) and leukemia-free survival (LFS) were 80.9% (95%CI, 63.5–93.6%) and 81.3% (95%CI, 64.2–93.7%), respectively. The 600-day relapse incidence (RI) and non-relapse mortality (NRM) was 6.9% (95%CI, 1.8–26.3%) and 11.7% (95%CI, 3.9–35.0%).

**Conclusion:**

Our study shows that the addition of venetoclax to a MAC allo-HSCT was feasible, safe and effective for high-risk AML patients.

## Introduction

Allogeneic hematopoietic stem cell transplantation (allo-HSCT) is the standard care option for acute myeloid leukemia (AML) patients classified as having an intermediate or high-risk karyotype by the National Comprehensive Cancer Network (NCCN) stratification system in any disease state such as first complete remission (CR1)/second complete remission (CR2) or above/active disease [1]. Allo-HSCT is also the standard therapy for those AML patients with favorable-risk disease, according to WHO risk stratification, who exhibit a poor molecular response to chemotherapy [1]. A phase III randomized trial that compared myeloablative conditioning (MAC) and RIC regimens in AML and myelodysplastic syndromes (MDS) showed that OS was higher with MAC, but these results were not statistically significant. RIC resulted in lower treatment-related mortality (TRM) but higher relapse rates compared with MAC, while the use of MAC resulted in a statistically significant advantage in relapse-free survival (RFS) compared to RIC [2]. These data support the use of MAC as the standard of care for fit patients with AML [2]. But even in the first remission, patients in the adverse risk group, as stratified according to the European Leukemia Net (ELN) risk classification, had the highest risk of relapse (HR, 1.47; *p* ≤ 0.001) and inferior disease-free survival (DFS; HR, 1.35; *p* < 0.001) and OS (HR, 1.39; *p* < 0.001) [3]. In AML patients younger than 50 years of age, the OS in the minimal residual disease (MRD)-negative group and MRD-positive group were 70.0 and 56.2%, respectively [4]. Data from CIBMTR show a 3-year OS for relapsed or primary induction failure (defined as failure to achieve remission following two cycles of induction chemotherapy) AML of only 19% [5]. According to the data from CIBMTR, although the TRM of allo-HSCT decreased over time, the risk of relapse increased, and as a result, treatment failure rates were relatively stable from 1980 to 2014 [6]. The survival rate of patients with AML aged 20-45 years old in CR1 with MAC after allo-HSCT did not significantly improve [6]. Currently, there are novel FDA-approved drugs and cellular therapies that have improved the clinical outcomes of AML. The population referred for transplantation most likely has disease that is biologically more aggressive and more difficult to cure regardless of the therapy used [6].

Venetoclax (ABT-199) is a BH3-mimetic agent [7]. BH3 mimetics act by inhibiting the pro-survival BCL-2 proteins to enable the activation of BAX and BAK, apoptosis effectors that permeabilize the outer mitochondrial membrane, triggering apoptosis directly in many cells and sensitizing others to cell death when combined with other antineoplastic drugs [8]. A phase II study in refractory/relapsed (R/R) AML patients has shown that venetoclax monotherapy can achieve an overall response rate (ORR) of 19% [9]. The ORR of venetoclax combined with decitabine-based treatment for heavily pre-treated R/R AML patients was 45.5% and the CR rate was 40.9% [10]. Venetoclax combined with fludarabine, cytarabine, granulocyte colony-stimulating factor and idarubicin (FLAG-IDA) achieved a high response rate in R/R AML [11]. Venetoclax may also target leukemic stem cells (LSCs) by specifically impairing amino acid-fueled oxidative phosphorylation [12,13]. When applied to non-myeloablative HSCT, the maximum tolerated dose (MTD) of venetoclax was not reached [14]. The 1-year OS and progression-free survival (PFS) was 67% (95% CI, 43–83%) and 53% (95% CI, 31–72%), respectively, for high-risk MDS and AML [14]. An optimal conditioning regimen that allows for a well-balanced antileukemia effect and limited toxicity is urgently needed. Therefore, here, in our current study, we evaluated the efficacy and safety of venetoclax as part of a MAC allo-HSCT for high-risk AML patients.

## Patients and methods

### Study design and enrolled patients

From 25 February 2021 to 4 September 2022, patients with AML who underwent venetoclax-containing MAC allo-HSCT at the Hebei Yanda Lu Daopei hospital were retrospectively analyzed. Patients eligible for enrolment had to meet the following criteria: (1) first transplantation, (2) venetoclax used in the conditioning regimens, (3) AML with high-risk features such as refractory or relapsed leukemia, MRD-positive at transplantation or adverse prognosis group according to ELN risk stratification [15,16]. The data cutoff date was 19 November 2022. This retrospective study was approved by the Ethics Committee of Hebei Yanda Lu Daopei Hospital.

### Gene mutation and fusion genes screening

Next generation sequencing (NGS) was performed on the ThermoFisher Ion Torrent PGM platform as described previously [17]. FLT3-ITD, NPM1 and CALR mutations were detected by fragment analysis (AB 3500XL sequencer), the results of which were analyzed using GeneMapper ID V3.2 software. A total of 131 mRNA isotypes of 41 fusion genes (Supplementary Tables 1 and 2), as well as MLL-PTD, IKZF1 and ERG deletion mutations were screened by multiplex-nested reverse-transcription PCR (RT-PCR) according to the protocols we previously reported [18,19].

### Myeloablative conditioning regimens

Initially, we utilized myeloablative conditioning regimens of venetoclax/decitabine/cytarabine followed by busulfan/fludarabine (Bu/Flu). When we found mixed chimerism of CD3 positive cells in some patients’ peripheral blood samples during the delivery of the Bu/Flu conditioning regimens, we switched to a venetoclax/fludarabine/cytarabine/G-CSF followed by busulfan/cyclophosphamide conditioning regimen for the some subsequently enrolled patients. Venetoclax was administered at −14 to −10 days for the following reasons: The terminal elimination half-life of venetoclax was approximately 26 h [20] and approximately 5½ days were required to clear the drug, so that it could be metabolized before transfusion of hematopoietic stem cells. This was necessary to avoid the effect on hematopoietic stem cells and a negative effect on implantation. In addition, we assumed that venetoclax is not cleared until after the Bu course was finished, potentially enhancing the anti-leukemic effect of Bu.

Venetoclax/decitabine/cytarabine consisted of venetoclax (400 mg/d oral for adult or 360 mg/m^2^/d oral for children [21]), decitabine (20 mg/m^2^/d IV) and cytarabine (2 g/m^2^/d IV) on day −14 to day −10. Detailed dosage of Bu/Flu was Bu 0.8 mg/kg IV Q6h for adults (see the Supplementary Table 3 for children), day −9 to day −6 and fludarabine 30 mg/m^2^ IV, day −5 to day −1. Semustine 250 mg/m^2^ was given orally on day −3. Venetoclax/fludarabine/cytarabine/G-CSF consisted of venetoclax (400 mg/d oral for adult or 360 mg/m^2^/d oral for children [21]), fludarabine (30 mg/m^2^/d IV), cytarabine (2 g/m^2^/d IV) and G-CSF 5 μg/kg (subcutaneous injection) on day −14 to day −10. Detailed dosage of Bu/Cy was Bu 0.8 mg/kg IV Q6h for adults (see the Supplementary Table 3 for children), day −9 to day −6 and cyclophosphamide 1 g/m^2^ day −5 to day −4. Semustine 250 mg/m^2^ was given orally on day −3. When combined with voriconazole or posaconazole, the dose of venetoclax was adjusted to 100 mg daily [22] for adult or 90 mg/m^2^/d for childern. Melphalan of 70 mg/m^2^ could be added to the conditioning regimen on day −1 for patients whose bone marrow MRD levels (measured by flow cytometry or fusion gene quantification) were elevated at the end of 5 days of venetoclax combination chemotherapy compared to the MRD level before transplantation.

### Graft-versus-host disease (GVHD) prophylaxis

Tacrolimus, mycophenolate mofetil, intravenous methotrexate (15 mg/m^2^ on day +1, then 10 mg/m^2^ on days +3, +6, and +11) and anti-human T lymphocyte immunoglobulin (ATG) were used for the prophylaxis of graft-versus-host (GVHD). The initial dose of tacrolimus was 0.015 mg/kg/day and was started on day −9 and continued intravenous infusion for 24 h. The dose of tacrolimus was adjusted according to the drug blood concentration. The total dose of ATG-F (formerly Fresenius, now Grafalon, Neovii Biotech GmbH) or ATG-P (anti-human T lymphocyte porcine immunoglubulin, Wuhan Institute of Biological Products Co., Ltd.) was 20 mg/kg. The total dose of ATG was divided over 4 days (from day −2 to day −5). ATG-F was used in haploidentical (haplo) HSCT and ATG-P was used for matched-sibling donor (MSD) HSCT.

### BCL-2 expression of leukemia cells and other laboratory test

The expression of the BCL-2 in leukemia cells were detected by flow cytometry (FCM) [23]. The ratio of the mean fluorescence intensity of BCL-2 on leukemia cells to the nucleated erythrocytes was used to estimate the expression intensity of BCL-2. The *C*_max_ of venetoclax (6 h after administration) [24] plasma concentration was determined by high-performance liquid chromatography tandem mass spectrometry. Bone marrow samples were collected on day −10 after administration of venetoclax/decitabine/cytarabine or venetoclax/fludarabine/cytarabine/G-CSF and a series times after neutrophil engraftment to evaluate MRD. MRD was detected by monitoring quantitative polymerase chain reaction (qPCR) to detect leukemia-related fusion genes and mutated genes such as NUP98::NSD1 and nucleophosmin gene (NPM1) and multiparameter FCM to detect leukemia associated immunophenotype (LAIP). The minimum sensitivities of gene markers and LAIPs to monitor MRD were 1 × 10^−5^ and 1 × 10^−3^ to 1 × 10^−4^, respectively. Donor-specific anti-HLA antibodies (DSA) were detected using a Luminex200 flow cytometer. Donor-recipient chimerism was analyzed by short tandem repeats (STR)-PCR with a sensitivity of 1 × 10^−2^. The CD3-positive cells isolated from peripheral blood cell were obtained by magnetic beads directly conjugated with an anti-CD3 monoclonal antibody. The 15 STR loci for chimerism analysis were D8S1179, D21S11, D7S820, CSF1PO, D3S1358, TH01, D13S317, D16S539, D2S1338, D19S433, vWA, TPOX, D18S51, D5S818 and FGA. We checked for and quantified plasma cytomegalovirus (CMV)-DNA and Epstein-Barr virus (EBV)-DNA until 100 days after transplantation by qPCR.

### Definitions

The impact of different comorbidities prior to transplant were evaluated by the HSCT specific comorbidity-index (HSCT-CI) [25]. Neutrophil engraftment was defined as the first 3 consecutive days with ANC ≥0.5 × 10^9^/L. Platelet engraftment was defined as the first 7 consecutive days with platelet counts ≥20 × 10^9^/L without infusion. Acute GVHD (aGVHD) was diagnosed and graded using previously published consensus criteria [26,27]. Chronic GVHD (cGVHD) was defined and graded according to the National Institutes of Health consensus criteria [28]. CR was defined as bone marrow blasts <5%, absence of circulating blasts and extramedullary disease, ANC ≥ 1.0 × 10^9^/L and platelet count ≥ 100 × 10^9^/L. CR with incomplete hematological recovery (CRi) was defined as meeting all CR criteria except for neutropenia (<1.0 × 10^9^/L) or thrombocytopenia (<100 × 10^9^/L) [15]. MRD-negative was defined as CR with negativity by qPCR and multiparameter FCM. OS was defined as the time from the first day of transplantation to death due to any cause or the last follow-up. Leukemia-free survival (LFS) was defined as the time from transplantation to relapse or death, or last follow-up. Relapse incidence (RI) was defined as the reappearance of blasts in blood, bone marrow (>5%) or any extramedullary site after achieving CR. Non-relapse mortality (NRM) was defined as death without relapse. Treatment related adverse events was evaluated from the first dose of venetoclax to the day of neutrophil engraftment. Adverse events were graded using the National Cancer Institute Common Toxicity Criteria for Adverse Events, version 5.0 (https://evs.nci.nih.gov/ftp1/CTCAE/About.html). Complete chimerism was defined as a recipient DNA (R-DNA) less than 1%, and mixed chimerism as the R-DNA percentage superior to this threshold.

### Statistical analysis

OS and LFS were analyzed by the Kaplan–Meier method using a Log-rank test. Cumulative incidence analysis of relapse, NRM, CMV, EBV, aGVHD and cGVHD were performed using Gray’s test, and death before the event of interest as a competing risk. A 2-side *p* < 0.05 was considered statistically significant. NCSS12 software was used for statistical analysis.

## Results

### Patient characteristics

At our center, 32 patients received a transplant with a conditioning regimen containing venetoclax. Patient 24 (P24) received a total body irridiation-based regimen and was excluded from the analysis. Therefore, a total of 31 patients were included in our retrospective analysis, including 12 males and 19 females. The median patient age was 25 years (range: 3–58 years). Twenty-five (80.6%) patients were in CR/CRi and 6 (19.4%) patients were in non-remission (NR) before transplantation. These 31 patients received a median of 3 (range: 1–9) cycles of chemotherapy prior to transplantation. Twenty (64.5%) had received 3 + 7 standard regimen, 8 (25.8%) a three-drug combination regimen, and 3 (9.7%) venetoclax combination chemotherapy as induction therapy. A total of 8 patients relapsed during chemotherapy. Five patients received re-induction therapy with venetoclax or selinexor combination chemotherapy and all of them proceed to transplantation after 4 patients achieved remission and 1 remained in no remission. The other 3 relapsed patients underwent transplantation directly without re-induction. Two refractory patients went to transplantation after three cycles of induction chemotherapy without remission.

**Figure 1.**
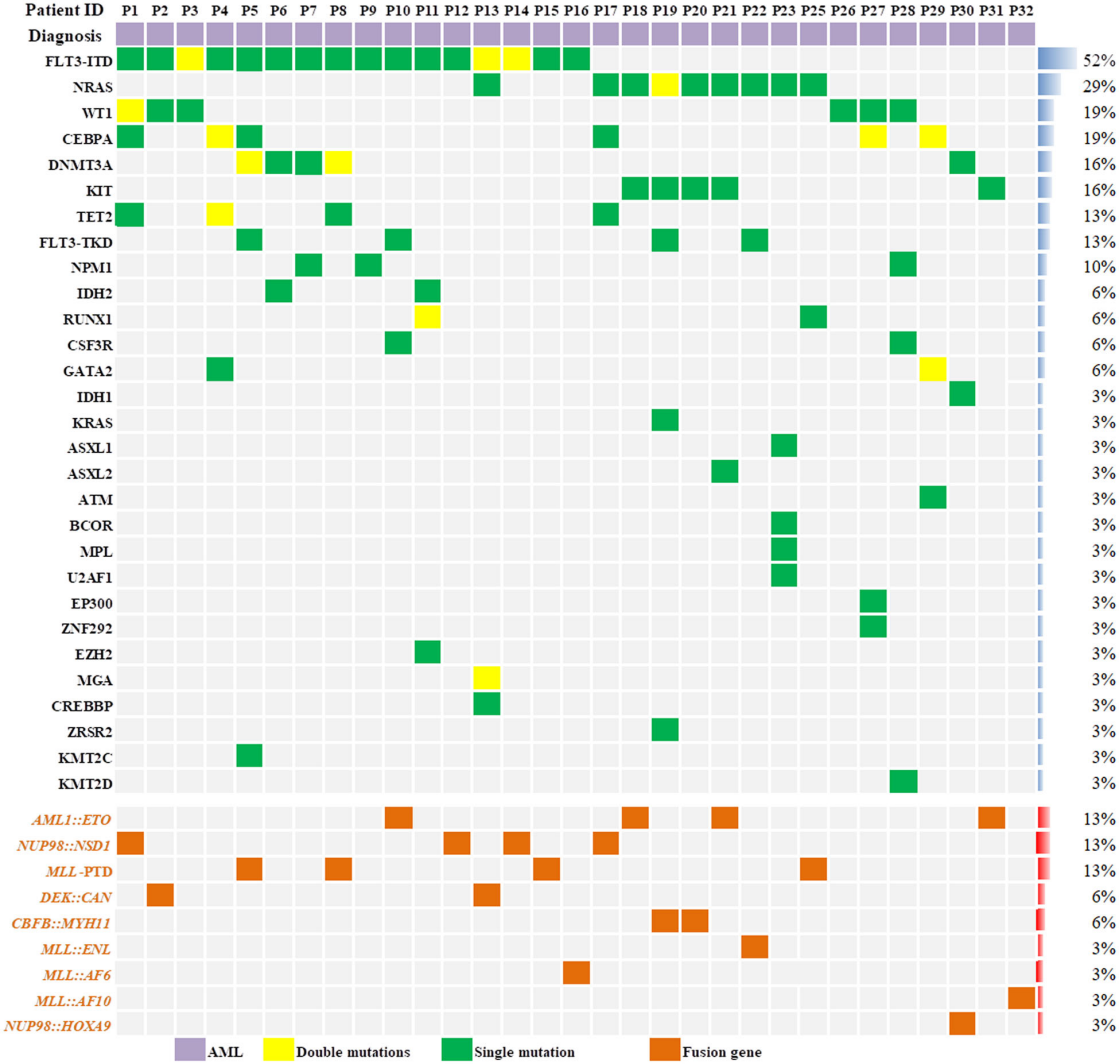
Details of the fusion genes and gene mutations among the 31 patients. The proportion of patients in the cohort with each alteration is reported on the right side of the figure. Columns represent individual patients, and rows represent clinical variables or the presence of gene mutations and fusion genes identified at diagnosis. The most common gene mutation among the 31 patients was FLT3-ITD. The most common fusion gene was the NUP98 rearrangement.

**Figure 2.**
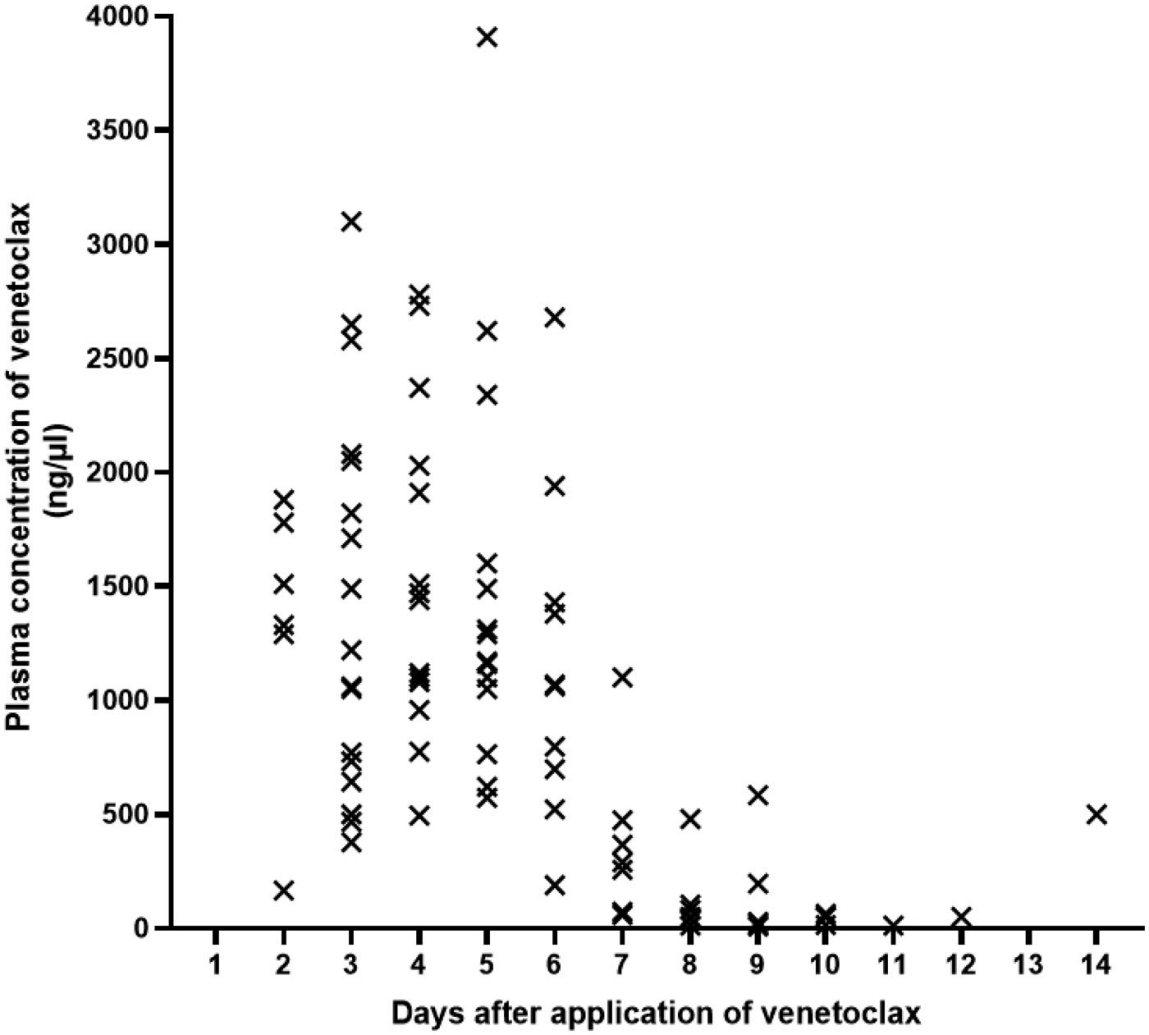
Plasma concentration of venetoclax. Venetoclax was administered orally daily for 5 days. Days 1–5 was C_max_. Days 6–14 was morning plasma concentrations after discontinuation of venetoclax.

Twenty-four patients (77.4%) were MRD-positive and 7 patients (22.6%) were MRD-negative before transplantation. The median white blood cell count at diagnosis was 23.4 (1.8–445.5)×10^9^/L. The details of the fusion genes and gene mutations are shown in Figure 1. The FLT3-ITD gene mutation was present in 51.6% of patients. NUP98 rearrangement, MLL rearrangement or MLL-PTD and DEK::CAN fusion genes were found in 5 (16.1%), 7 (22.6%) and 2 (6.5%) patients, respectively. Three patients with AML1::ETO fusion gene-positive AML had a KIT mutation and one of the three patients had systemic mastocytosis. The fourth AML1::ETO fusion gene positive AML patient was in CR2 prior to transplantation. One AML patient achieved CR1 after 4 cycles of chemotherapy combined with SKLB1028 (an oral multi-kinase inhibitor of EGFR, FLT3 and Abl) [29]. The expression of BCL-2 in leukemia cells was detected by FCM in 23 of the 31 patients. Leukemia cells from all 23 patients expressed BCL-2 and median percentage of BCL-2 expression in leukemia cells was 92.7% (range: 15.1–98.0). The median ratio of the mean fluorescence intensity (MFI) of BCL-2 level in leukemia cells relative to nucleated erythrocytes was 11.7-fold higher (range: 3.0–23.2). Two patients had extramedullary involvements at transplantation. The median leukocyte, hemoglobin and platelet count were 2.5 × 10^9^/L (range: 0.1–10.1), 86.7 g/L (range: 7.6–142.7), and 127.7 × 10^9^/L (range: 9.9–445.2) at initiation of conditioning, respectively. DSA was detected in 29 patients. Twenty-one patients were negative, 5 patients were positive and 2 patient was strongly positive for DSA. One patient was given bortezomib, rituximab and plasma exchange for the removal of DSA prior to transplantation. The other patient was not treated for DSA because the donor was changed during the conditioning regimen course. Plasma concentrations of venetoclax were measured in 28 patients. Median *C*_max_ of venetoclax on the second, third, fourth and fifth days after oral administration of venetoclax were 1420 ng/μl (range: 166–1880), 1220 ng/μl (range: 377–3100), 1455 ng/μl (range：494–2780) and 1230 ng/μl (range 573–3910), respectively. On the first day after stopping venetoclax, the median plasma concentration of venetoclax was still 1065 ng/μl (range：189–2680). From the second day after the discontinuation of venetoclax, the plasma concentration of venetoclax began to decrease significantly (Figure 2). Detailed patient characteristics are showed in Table 1.

**Table 1.** Patient, disease, and transplant characteristics (*n* = 31).

Characteristic	Value
Age of patients, yr, median(range)	25 (3–58)
Gender, *n* (%)	
Female	19 (61.3)
male	12 (38.7)
HCT-CI scores, *n* (%)	
0	12 (38.7)
1	15 (48.4)
2	2 (6.5)
3	2 (6.5)
ECOG performance status scale before HSCT, *n* (%)	
0	2 (6.5)
1	19 (61.3)
2	7 (22.6)
3	3 (9.7)
Diagnosis, *n* (%)	
De novo AML	28 (90.3)
MDS transformed AML	3 (9.7)
White blood count at diagnosis, median(range) ×10^9^/L	23.4 (1.8–445.5)
Fusion genes at diagnosis, *n* (%)	
AML1::ETO	4 (12.9)
CBFβ::MYH11	2 (6.5)
DEK::CAN	2 (6.5)
MLL::AF10	1 (3.2)
MLL::AF6	1 (3.2)
MLL::ENL	1 (3.2)
MLL-PTD	4 (12.9)
NUP98::HOXA9	1 (3.2)
NUP98::NSD1	4 (12.9)
Negative	11 (35.5)
Number of chemotherapy cycles required to achieve first CR, *n* (%)	
1	16 (51.6)
2	6 (19.4)
≥3 or never achieved CR	9 (29.0)
Disease status pre-HSCT; *n* (%)	
CR1	21 (67.7)
CR2	4 (12.9)
relapse/refractory	6 (19.4)
MRD pre-HSCT, *n* (%)	
Negative	7 (22.6)
Positive	24 (77.4)
Donor type, *n* (%)	
MSD	2 (6.5)
Haploidentical	29 (93.6)
ABO compatibility, *n* (%)	
Matched	14 (45.2)
Major mismatched	7 (22.6)
Minor mismatched	8 (25.8)
Bidirectional	2 (6.5)
CMV serologies (donor/recipient), *n* (%)	
Positive/positive	29 (93.5)
Negative/positive	2 (6.5)
EBV serologies (donor/recipient), *n* (%)	
Positive/positive	31 (100)
Conditioning regimen, *n* (%)	
Venetoclax/Decitabine/Ara-C/Bu/Flu	20 (64.5)
Venetoclax/Decitabine/Ara-C/Bu/Flu/Mel	6 (19.4)
Venetoclax/FLAG/Bu/Cy	5 (16.1)
GVHD prophylaxis, n (%)	
Tacrolimus + MMF + MTX	31 (100)
Antithymocyte globulin (ATG), *n* (%)	
ATG-F	29 (93.5)
ATG-P	2 (6.5)
Number of infused MNC; median(range)×10^8^/kg	9.2 (3.9–15.2)
Number of infused CD34+ cells; median(range)×10^6^/kg	4.0 (3.8–7.5)
Number of infused CD3+ cells; median(range)×10^8^/kg	1.9 (0.4–9.8)

AML: acute myeloid leukemia; HCT-CI: hematopoietic cell transplantation-comorbidity index; MDS: myelodysplastic syndromes; CR: complete remission; MRD: minimal residual disease; MSD-HSCT: matched sibling donor hematopoietic cell transplantation; Haplo-HSCT: haploidentical hematopoietic stem cell transplantation; CMV: cytomegalovirus; EBV: Epstein–Barr virus; FLAG: fludarabine/cytarabine/granulocyte-colony stimulating factor; Bu: busulfan; Flu: fludarabine; Mel: melphalan; Cy: cyclophosphamide; ATG-F: ATG-Fresenius; ATG-P: ATG-porcine; MNC: mononuclear cells.

### Engraftment

The median time of neutrophil and platelet engraftment was 12 days (range: 10–25) and 14 days (range: 10–60), respectively. The 30-day cumulative incidence of neutrophil engraftment was 100%. The 30-day cumulative incidence of platelet engraftment was 93.6% (95%CI, 85.3–100%). The 60-day cumulative incidence of platelet engraftment was 96.8% (95%CI, 90.8–100%).

### MRD monitoring

**Figure 3.**
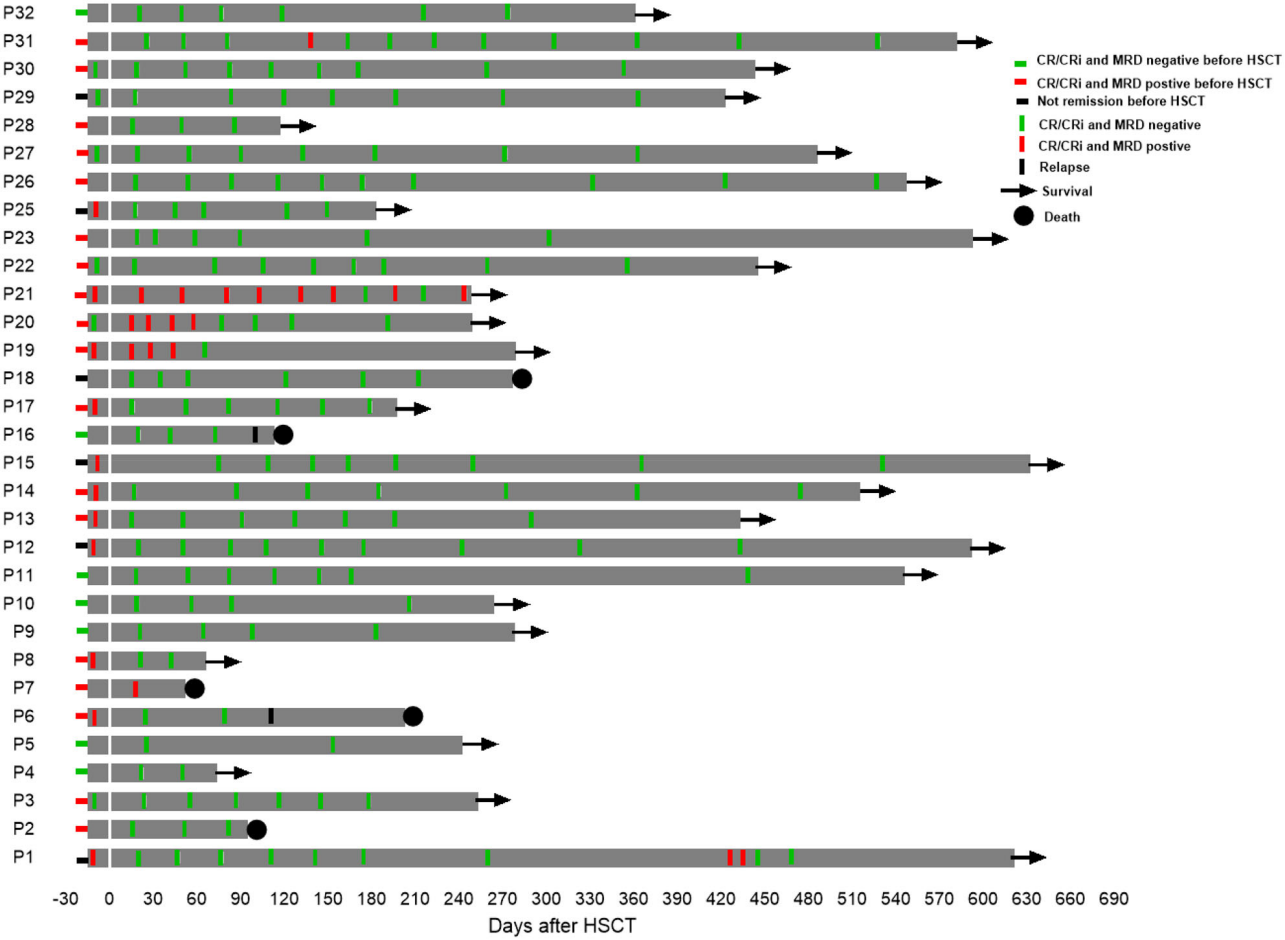
Serial minimal residual disease (MRD) monitoring before and after transplant. MRD was detected by flow cytometry and gene quantification, and a negative result using both methods was defined as negative. Each bar represents a patient, with the length of the gray bars indicating follow-up time. Symbols indicate MRD, relapse or death at serial time points.

Bone marrow aspirates were performed on day −10 to evaluate MRD status after the administration of venetoclax plus chemotherapy in the 19 MRD-positive patients (3 in non-remission at transplantation). MRD was elevated in 7 patients. In 6 of these cases, melphalan was added to the conditioning regimen. One female patient was not given melphalan as she was experiencing grade 3 diarrhea. Of the 6 cases of core binding factor AML, 2 patients were negative and 4 patients were positive for fusion gene by RT-PCR after HSCT. One patient (P21) had AML1::ETO positive with system mastocytosis and showed the presence of persistent low-copy AML::ETO fusion gene (range: 0.00438–0.00017) post transplantation even after administration of midostaurin and avapritinib. In the remaining three core binding factor AML patients whose fusion gene were positive after transplant, MRD status turned negative after the dose of immunosuppressive drugs was reduced or the patient received midostaurin, avapritinib or chidamide after transplantation. One patient (P7) with the NPM and FLT3-ITD gene mutations remained NPM gene positive at the first post-transplant bone puncture, and the patient died from GVHD at 52 day after transplantation. The MRD status of one NUP98::NSD1 positive AML patient (P1) became positive at day 427 after transplantation and then the patient became MRD negative after receiving low dose of chemotherapy and donor lymphocyte infusion (DLI). Two patients experienced relapse and death at follow-up time (P6 and P16). The remaining 23 evaluable patients achieved persistent MRD-negative status after transplantation (Figure 3).

### Chimerism analysis

**Figure 4.**
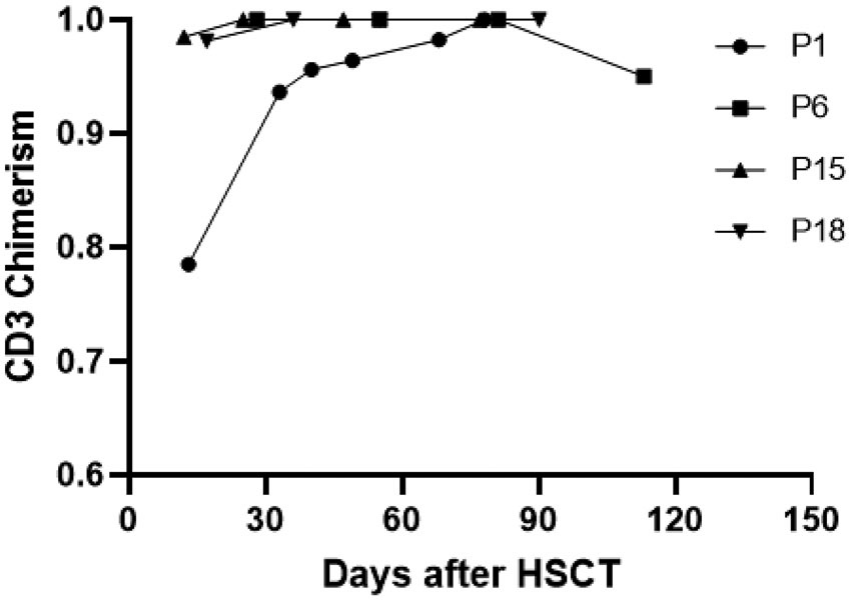
Changing chimerism rate trends of CD3 positive cells in peripheral blood. Patient 1(P1) had DLI on day 25 post-transplant at a dose of 1 × 10^7^/kg, resulting in a final chimerism rate of 100%. Patients 15 and 18 achieved 100% chimerism after reduction in the dose of immunosuppressants. Patient 6 experienced a decrease in peripheral blood CD3+ cells chimerism 112 days after transplantation. This patient later died of relapse.

The chimerism of CD3 positive cells in peripheral blood was evaluated in all 31 patients, and CD3 positive cell of 27 patients were 100% donor-type. The other 4 patients had mixed chimerism at some time point following transplantation (Figure 4). The chimerism rate of CD3 cells in peripheral blood of one patient (P1) was 78.5% at +13 days after transplantation and the chimerism rate reached 100% after receiving DLI. The chimerism rate of 2 patients reached 100% after reducing the dose of immunosuppressant. One patient (P6) relapsed on day +112 and the chimerism rate of peripheral blood CD3 positive cells reduced from 100 to 95.01%. The bone marrow cells of all evaluated patients showed complete chimerism except for the relapsed patients.

### Acute and chronic GVHD

The 100-day incidence of grade 2 to 4 aGVHD was 64.5% (95%CI, 49.7–83.8%), and the incidence of grade 3 to 4 aGVHD was 16.1% (95%CI, 7.2–36.0%). The incidence of all cGVHD at 180 days was 42.5% (95%CI,27.6–65.4%). The 180-day cumulative incidence of moderate to severe cGVHD was 7.1% (95%CI, 1.9–26.9%).

### Viral infection-related complications and adverse events

Cumulative incidence of 100-day CMV viraemia and 100-day EBV viraemia was 61.6% (95%CI, 46.5–81.4%) and 3.2% (95%CI, 0.5–22.2%), respectively. The treatment related adverse events were assessed at −14 days from pre-transplantation conditioning to the neutrophil engraftment. The treatment related adverse events are listed in the Table 2. The most common grade 3 or above adverse events were cytopenia and infection.

**Table 2.** Summary of treatment-related adverse events according to CTCAE, version5.0.

AEs	Grade1-2, *n* (%)	Grade 3, *n* (%)	Grade 4, *n* (%)
Hemoglobin	–	31 (100)	–
Neutropenia	–	31 (100)	–
Thrombocytopenia	–	31 (100)	–
Coagulation	4 (12.9)	–	–
Cardiac disorders	31 (100)	–	–
Ear	2 (6.5)	–	–
Thyroid	–	–	–
Eye	3 (9.7)	–	–
Diarrhea	17 (54.8)	3 (9.7)	3 (9.7)
Oral mucosa	6 (19.4)	11 (33.3)	–
Edema of limbs	1 (3.2)	–	–
Fatigue	31 (100)		–
Gallbladder	–	–	–
Liver	10 (32.3)	–	–
Hepatic portal system	–	–	–
Allergy	1 (3.2)	7 (22.6)	–
CRS	–	–	–
Bacterial and fungal Infections	18 (58.1)	13 (41.9)	
Vein	2 (6.5)	–	–
Artery	–	–	–
Electrolyte	31 (100)	–	–
Iron overload	–	–	–
Central nervous system	–	–	–
Peripheral nervous system	1 (3.2)	–	–
Psychiatric disorders	–	–	–
Renal and urinary disorders	3 (9.7)	–	–
Reproductive system	–	–	–
Respiratory disorders	5 (16.1)	–	–
Skin and subcutaneous tissue disorders	31 (100)	–	–

CTCAE: common toxicity criteria for adverse events; CRS: cytokine release syndrome.

### Survival

**Figure 5.**
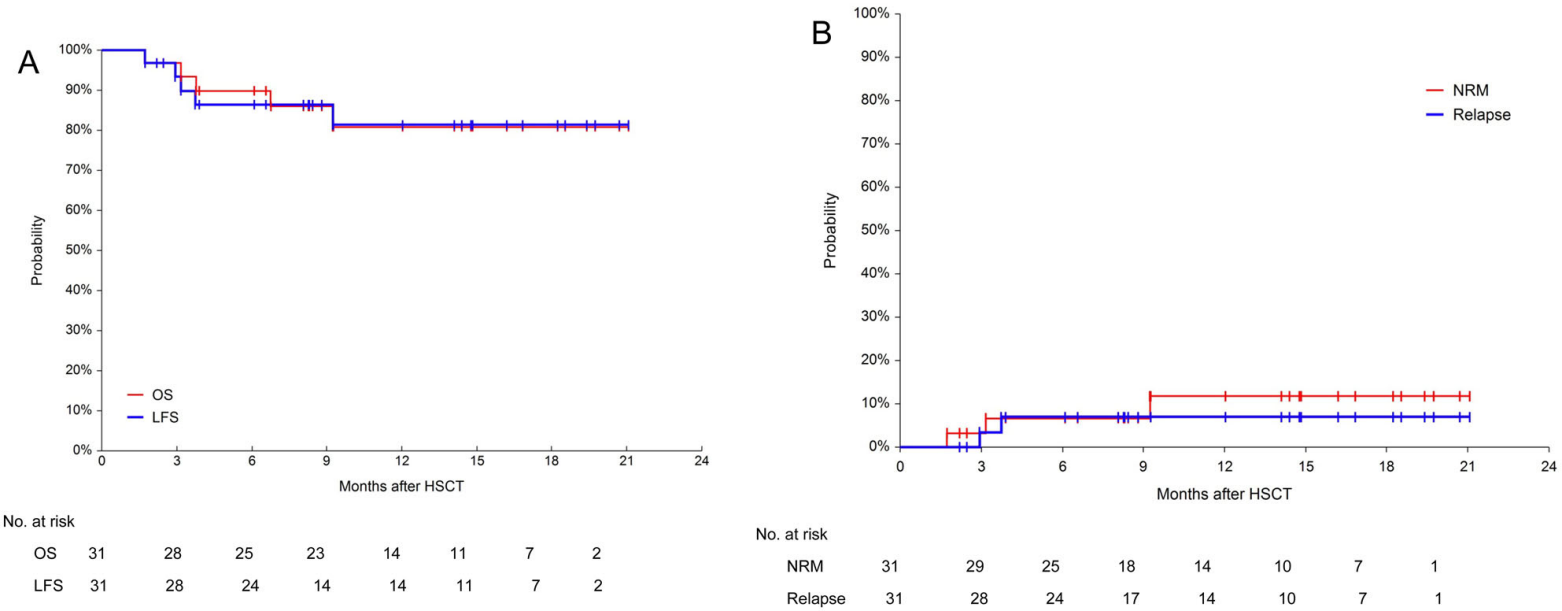
Overall survival(OS), leukemia-free survival(LFS), non-relapse mortality(NRM) and relapse incidence(RI) for the 31 patients. (A) Probabilities of OS and LFS and (B) Probabilities of NRM and RI.

**Figure 6.**
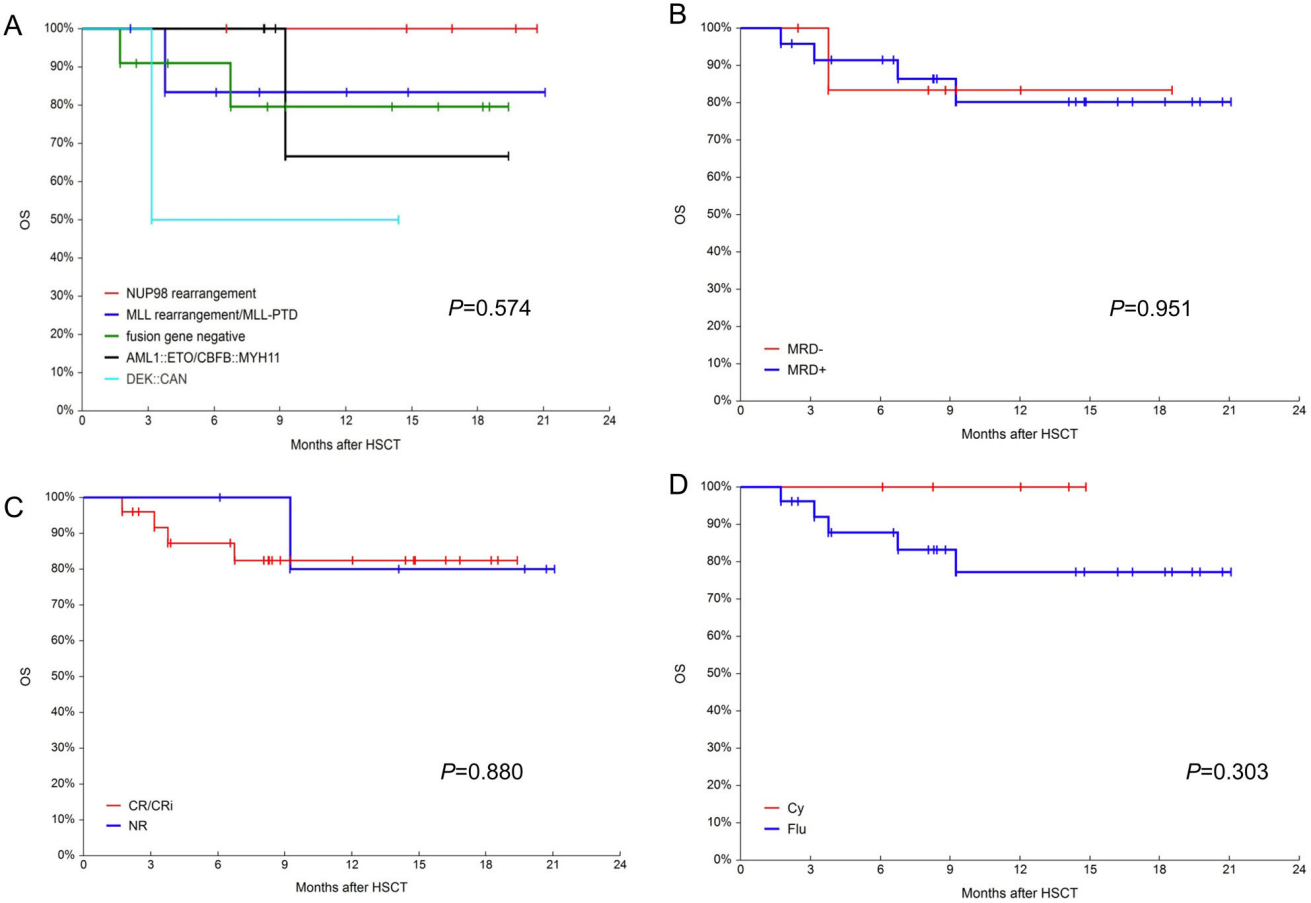
OS in different groups. (A) Probabilities OS in different fusion genes groups; (B) probabilities OS in MRD negative and positive groups; (C) probabilities OS in CR/CRi and NR groups and (D) probabilities OS in busulfan/ cyclophosphamide(Bu/Cy) and busulfan/fludarabine(Bu/Flu) conditioning regimens groups.

The median follow-up time was 278 days (range: 52–632). The 600-day OS and LFS were 80.9% (95%CI, 63.5–93.6%) and 81.3% (95%CI, 64.2–93.8%), respectively. The 600-day RI was 6.9% (95%CI, 1.8–26.3%). The 600-day NRM was 11.7% (95%CI, 3.9–35.0%) (Figure 5). The 1-year OS in the NUP98 rearrangement, MLL rearrangement/MLL-PTD, fusion gene negative, core binding factor AML and DEK::CAN groups were 100%, 83.3% (95% CI, 46.5–100%), 79.6 (95%CI, 49.9–97.7%), 66.7% (95%CI,14.4–99.8%) and 50.0% (95%CI, 0.9–99.2%) (*p* = 0.574), respectively. Given the small number of patients, the *p*-values were not statistically different, but a trend could be seen (Figure 6(A)). All patients with NUP98 rearrangement survived. In the case of core binding factor AML, most of these patients required post-transplant interventions before their MRD status became negative. The 1-year OS was 83.3% (95CI, 46.5–100%) and 80.2% (95%CI, 60.1–94.4%) for the pre-transplant MRD-negative and MRD-positive groups, respectively (*p* = 0.951, Figure 6(B)). The 1-year OS was 82.4% (95% CI, 64.4–95.0%) and 80.0% (95% CI, 38.5–99.9%) in the CR/CRi and NR groups, respectively (Figure 6(C)). The 1-year OS for patients who underwent cyclophosphamide vs. fludarabine based conditioning regimen were 100 and 77.23% (95% CI, 57.3–92.2%), respectively (*p* = 0.303, Figure 6(D)). During the follow-up, 5 patients died. One patient was diagnosed with pulmonary tuberculosis and gave up treatment for personal reasons and later died from relapse. Another patient, who received SKLB1028 before transplantation, died from transplant-associated thrombotic microangiopathy (TA-TMA). The remaining 3 patients died from infectious shock, GVHD and relapse, respectively.

## Discussion

Although TRM has declined steadily over recent decades, no real progress has been made in reducing the risk of AML disease recurrence, which remains the major cause of transplant failure and an area of significant unmet need [30]. The outcomes of MAC HSCT for high-risk, MRD-positive or R/R AML patients remain unsatisfactory [3–5]. We need new transplant protocols to improve outcomes for high-risk AML patients [16]. Garcia J S et al. has reported outcomes of patients receiving venetoclax as part of a RIC transplant [14]. To our knowledge, here, we report the first use of venetoclax within a myeloablative conditioning regimen for high-risk AML patients who underwent allo-HSCT. Our preliminary results show a 600-day OS and LFS of 80.9 and 81.3%, and a 600-day RI and NRM of 6.9 and 11.7%, respectively. Although extended follow-up time is required, considering that 77.4% of patients had a positive MRD status and 19.4% of patients had R/R AML before transplantation, the clinical outcomes are encouraging. A meta-analysis of 1830 patients from 15 clinical trials showed that NRM at 100 days was lower in the Bu/Flu conditioning regimen group compared to the Bu/Cy group (relative risk 0.56; 95% CI 0.34–0.92) [31]. Of the 31 patients on our study, 26 patients underwent Bu/Flu conditioning regimen, while 5 patients underwent a Bu/Cy conditioning regimen although cyclophosphamide was reduced from the conventional 60 mg/kg per day to 1 g/m2 per day, which could also explain the low NRM in our cohort.

Venetoclax monotherapy [9] or in combination with HMA [32] or other cytotoxic drugs such as FLAG-IDA [11], or CLAG [33] could achieve high response rates in newly diagnosed or R/R AML. However, combinations of venetoclax and Bu, 4-hydroperoxycyclophosphamide or fludarabine has synergistic cytotoxicity [34]. The first day after the last venetoclax dose, the median plasma concentration of venetoclax remained at 1065 ng/μl (range：189–2680). Even though the concentration of venetoclax decreased significantly from the second day and going forward, the venetoclax concentration remained at detectable levels during Bu infusion. It is possible that venetoclax may still have a synergistic effect with Bu in this context.

We stratified patients according to their MRD response to venetoclax plus chemotherapy as part of their conditioning regimen. Melphalan was added on day −1 for patients with elevated MRD levels to improve clearance of leukemic cells. This also avoids increasing conditioning regimen intensity in chemotherapy-sensitive patients which may increases transplant-related toxicity. Overexpression of BCL-2, BCL-xl, and MCL1 frequently occurs in AML, conferring resistance to conventional chemotherapy [7]. Expression levels of BCL-2 are correlated with sensitivity to venetoclax [35]. BCL-2 expression is high in AML, including in leukemia stem cells (LSCs) [12]. In our study, BCL2 expression was detected by FCM in 23 patients and all 23 patients had strong BCL2 expression compared to control nucleated erythrocytes. This strong BCL2 expression may be one of the reasons for the strong anti-tumor effect of venetoclax-containing conditioning regimens.

NUP98::rearranged AML are now recognized as a high-risk subtype of leukemia [36], and NUP98-NSD1 was found in 15% of FLT3/ITD and 7% of cytogenetically normal (CN)-AML. Those with dual FLT3/ITD and NUP98::NSD1 (82% of NUP98::NSD1 positive patients) had a CR rate of 27 vs. 69% among those patients with FLT3/ITD but without the NUP98::NSD1 fusion (*p* < 0.001). The corresponding 3-year OS was 31 vs. 48% (*p* = 0.011), respectively [37]. NUP98::HOXA9 fusion AML had a median OS of 13.5 months [38]. In our study, 4 patients were positive for the NUP98::SD1 fusion gene and 1 patient was positive for the NUP98::HOXA9 fusion gene. Two patients were refractory to chemotherapy and 3 patients were MRD-positive prior to transplantation. During the follow-up, all patients remained alive. Median OS was significantly shorter for AML patients harboring MLL-PTD and FLT3-ITD mutations [39]. The MLL(KMT2A) rearrangement was associated with adverse prognosis regardless of translocation subtype [40]. Allo-HSCT in CR1 was associated with improved OS (52% at 5 years vs 14% for those without allo-HSCT, *p* < 0.0001) [40]. The 5-year OS was 42% and the 5-year RI was 37% (*n* = 426) for patients with the MLL rearrangement post-HSCT in the CIBMTR database [41]. The 1-year OS for the 7 patients (2 patients in NR before transplantation) with the MLL rearrangement or MLL-PTD was 83.3% (95% CI, 46.5–100%). These results suggest that the MAC regimen containing venetoclax is particularly effective for AML patients with NUP98 rearranged or MLL-PTD disease.

The 100-day incidence of grade 3–4 aGVHD was 16.1% (95%CI, 7.2 − 36.0%) in our study. The cumulative incidences of grades 3–4 aGVHD for patients who underwent MSD HSCT and those treated with haplo-HSCT were comparable (7 vs. 3%, *p* = 0.173) [42]. Wang et al. reported the cumulative incidences for grades 3–4 aGVHD at 100 days of 10% (95%CI, 6–14%) and 3% (95% CI, 1–5%) after haplo and MSD HSCT for AML, respectively (*p* = 0.004) [43]. From the limited data available in this study, it appears that a proportion of patients still had detectable venetoclax in their plasma the day before the stem cell infusion, so an effect of venetoclax on T cells in the grafts cannot be ruled out. Relevant studies [44] have confirmed that venetoclax does not induce T-cell apoptosis compared to the vehicle control, even at the highest concentrations. BCL-2 expression was significantly higher in activated B cells compared to T cells, explaining the differential effect of the BCL-2 inhibitor on activated T and B cells [44]. Venetoclax can increase T cell effector function by increasing reactive oxygen species (ROS) generation without inducing T cell apoptosis [45]. Thus, the relatively high 3–4 grade aGVHD in this cohort of patients may be due to the addition of venetoclax to the conditioning regimen.

In summary, our study showed that adding venetoclax to a MAC regimen of allo-HSCT was feasible, safe and effective for high-risk AML. Our analysis is limited by the retrospective nature and relatively short follow-up. More data and longer observation time are needed to further evaluate the efficacy of venetoclax as part of this regimen. Studies to figure out which patients would benefit from this conditioning regimen are also needed, although this will be a challenging undertaking.

## Supplementary Material

Supplemental Material
