# Supplementary material for: Addition of venetoclax to myeloablative conditioning regimens for allogeneic hematopoietic stem cell transplantation in high-risk AML
Source: Ann Med. 2023 Jan 11;55(1):388–400. doi: 10.1080/07853890.2022.2164610 (PMC9851264; doi:10.1080/07853890.2022.2164610)
Supplement: Supplemental Material [file IANN_A_2164610_SM9445.doc]

**Supplementary Table 1. Genes and sequencing regions in the 90-**gene panel

| **Gene** | **Detection range** | **Gene** | **Detection range** |
| --- | --- | --- | --- |
| ALK | Exon 23-25 | JAK3 | Exon 11-16 |
| ANKRD26 | 5’UTR | KDM5C | Exon 1-26 |
| ASXL1 | Exon 13 | KDM6A | Exon 23-26 |
| ASXL2 | Exon 11-12 | KIT | Exon 8, 11, 17 |
| ATM | Exon 2-63 | KMT2C | Exon 1-58 |
| B2M | Exon 1-3 | KMT2D | Exon 1-54 |
| BCL2 | Exon 2-3 | KRAS | Exon 2-5 |
| BCL6 | Exon 1 | MEF2B | Exon 2-3 |
| BCOR | Exon 1-14 | MGA | Exon 2-23 |
| BIRC3 | Exon 6-9 | MPL | Exon10 |
| BRAF | Exon 15 | MYD88 | Exon 5 |
| CALR | Exon 9 | NF1 | Exon 1-58 |
| CARD11 | Exon 5-9 | NOTCH1 | Exon 26-27，Exon 34 |
| CBL | Exon 1-16 | NOTCH2 | Exon 34 |
| CCND1 | Exon 1 | NPM1 | Exon 11 |
| CCND3 | Exon 5 | NRAS | Exon 2-5 |
| CD28 | Exon 1-4 | NT5C2 | Exon 11，15 |
| CD58 | Exon 1-6 | PAX5 | Exon 1-10 |
| CD79B | Exon 5-6 | PHF6 | Exon 2-10 |
| CDKN1B | Exon 1-2 | PIGA | Exon 2-6 |
| CEBPA | Exon 1 | PIK3CA | Exon 10，21 |
| CREBBP | Exon 25-27 | POT1 | Exon 5-11 |
| CRLF2 | Exon 6 | PTEN | Exon 1-9 |
| CSF3R | Exon 14, 17 | PTPN11 | Exon 2-15 |
| CXCR4 | Exon 2 | RHOA | Exon 2 |
| DDX41 | Exon 1-17 | RUNX1 | Exon 2-9 |
| DNMT3A | Exon 19-23 | SETBP1 | Exon 4(c.2482_2813) |
| EGFR | Exon18-21 | SF3B1 | Exon 14-16 |
| EP300 | Exon 23-30 | SH2B3 | Exon 2-8 |
| ETNK1 | Exon 3 | SRP72 | Exon 1-19 |
| ETV6 | Exon 1-8 | SRSF2 | Exon 1(c.187_362) |
| EZH2 | Exon 2-20 | STAT3 | Exon 21 |
| FBXW7 | Exon 9-10 | STAT5B | Exon 15-16 |
| FLT3 | Exon 14-15, 20 | STAT6 | Exon 12-13,17 |
| FOXO1 | Exon 1 | TCF3(E2A) | Exon 17 |
| GATA1 | Exon 2-6 | TET2 | Exon 3-11 |
| GATA2 | Exon 2-6 | TNFAIP3(A20) | Exon 2-9 |
| GATA3 | Exon 4-5 | TNFRSF14 | Exon 1-3 |
| GNA13 | Exon 1-4 | TP53 | Exon 3-11 |
| ID3 | Exon 1-2 | U2AF1 | Exon 2, 6 |
| IDH1 | Exon 4(c.145_414) | WHSC1 | Exon 18-19 |
| IDH2 | Exon 4 | WT1 | Exon 7-9 |
| IL7R | Exon 6 | XPO1 | Exon 15 |
| JAK1 | Exon 14，18，19 | ZNF292 | Exon 1-8 |
| JAK2 | Exon 14(V617F) | ZRSR2 | Exon 1-11 |

**Supplementary Table** 2. 41 fusion genes included in our screening panel

| 1. BCR::ABL1 | 2. BCR::FGFR1 | 3. BCR::JAK2 | 4. CBFA2T3::GLIS2 |
| --- | --- | --- | --- |
| 5. CBFB::MYH11 | 6. DEK::NUP214(DEK::CAN) | 7. EBF1::PDGFRB | 8. ETV6::ABL1 |
| 9. ETV6::PDGFRB | 10. ETV6::RUNX1 | 11. FIP1L1::PDGFRA | 12. FUS::ERG |
| 13. KMT2A::AFDN(MLL::AF6) | 14. KMT2A::AFF1 | 15. KMT2A::ELL | 16. KMT2A::EPS15 |
| 17. KMT2A::MLLT1(MLL::ENL) | 18. KMT2A::MLLT10(MLL::AF10) | 19. KMT2A::MLLT11 | 20. KMT2A::MLLT3 |
| 21. KMT2A::MLLT6 | 22. KMT2A::MLLT7 | 23. NPM1::ALK | 24. NPM1::MLF1 |
| 25. NPM1::RARA | 26. NUP98::HOXA9 | 27. NUP98::HOXD13 | 28. NUP98::NSD1 |
| 29. PAX5::JAK2 | 30. PCM1::JAK2 | 31. PML::RARA | 32. RUNX1::EAP |
| 33. RUNX1::MECOM | 34. RUNX1::RUNX1T1(AML1::ETO) | 35. SET::NUP214 | 36. STIL::TAL1 |
| 37. TCF3::HLF | 38. TCF3::PBX1 | 39. TCF3::ZNF384 | 40. ZBTB16::RARA |
| 41. ZMYM2::FGFR1 |  |  |  |

**Supplementary Table 3. Dosage of Bu for children**

| Body weight | ＜9kg | 9-16kg | 16-23kg | ＞23-34kg | ＞34kg |
| --- | --- | --- | --- | --- | --- |
| Dosage of Bu (Q6h) | 1.0mg/kg | 1.2mg/kg | 1.1mg/kg | 0.95mg/kg | 0.8mg/kg |
